# The Clinical Value of Neutrophil-to-Lymphocyte Ratio and Platelet-to-Lymphocyte Ratio for Predicting Hematoma Expansion and Poor Outcomes in Patients with Acute Intracerebral Hemorrhage

**DOI:** 10.3390/jcm12083004

**Published:** 2023-04-20

**Authors:** Yejin Kim, Jong-Hee Sohn, Chulho Kim, So Young Park, Sang-Hwa Lee

**Affiliations:** 1 Institute of New Frontier Research Team, Hallym University, Chuncheon 24252, Republic of Korea; 2 Department of Neurology, Hallym University Chuncheon Sacred Heart Hospital, Chuncheon 24252, Republic of Korea; 3 Department of Endocrinology and Metabolism, Kyung Hee University Hospital, Seoul 02447, Republic of Korea

**Keywords:** NLR, PLR, inflammatory marker, hematoma expansion, intracerebral hemorrhage

## Abstract

There is little knowledge of the effect of inflammatory markers on the prognoses of hematoma expansion (HE) in patients with intracranial hemorrhage (ICH). We evaluated the impact of neutrophil–lymphocyte ratio (NLR) and platelet–lymphocyte ratio (PLR) on HE and worse outcomes after acute ICH. This study included 520 consecutive patients with ICH from the registry database enrolled over 80 months. Patients’ whole blood samples were collected upon arrival in the emergency department. Brain computed tomography scans were performed during hospitalization and repeated at 24 h and 72 h. The primary outcome measure was HE, defined as relative growth >33% or absolute growth <6 mL. A total of 520 patients were enrolled in this study. Multivariate analysis showed that NLR and PLR were associated with HE (NLR: odds ratio [OR], [95% CI] = 1.19 [1.12–1.27], *p* < 0.001; PLR: OR, [95% CI] = 1.01 [1.00–1.02], *p* = 0.04). Receiver operating characteristic curve analysis revealed that NLR and PLR could predict HE (AUC of NLR: 0.84, 95% CI [0.80–0.88], *p* < 0.001; AUC of PLR: 0.75 95% CI [0.70–0.80], *p* < 0.001). The cut-off value of NLR for predicting HE was 5.63, and that of PLR was 23.4. Higher NLR and PLR values increase HE risk in patients with ICH. NLR and PLR were reliable for predicting HE after ICH.

## 1. Introduction

Patients (about 40%) with spontaneous intracerebral hemorrhage (ICH) could experience death within 1 month after ICH. Only 39% of survivors achieve long-term independent functional status [1,2]. Early neurological deterioration via hematoma expansion (HE) could occur in 20–40% of patients with ICH and contributes to a high rate of disability and short-term mortality in patients with ICH [3]. Efforts have been made to predict the risk factors for HE and to identify patients with ICH at high risk of developing HE; however, there is no suitable marker or model for predicting HE after developing ICH. Since HE occurs after ICH, the management is extremely limited, and the prognosis could be devastating. Early detection of high-risk patients is important in acute stroke settings [4].

Among several predictors of prognosis for ICH [5], the inflammatory reaction could play a crucial role in propagating brain injury via aggravation and formation of brain swelling after ICH [6,7]. We speculated that since thrombin activation and the inflammatory cascade alter the permeability of the blood–brain barrier, acute inflammatory reactions could affect HE after ICH [8]. Although emerging evidence has shown that inflammatory markers could be useful [9,10,11], reliable methods for predicting early HE after ICH are lacking.

The neutrophil-to-lymphocyte ratio (NLR) and platelet-to-lymphocyte ratio (PLR) are fast and easy to obtain, and they assess systemic inflammation in the acute stage of ICH. Several studies have reported that NLR and PLR could indicate worse outcomes after ICH [9,10,11,12,13]. However, little is known about whether systemic inflammatory markers measured on hospitalization using NLR and PLR values correlate with early HE after ICH. In this study, we evaluated the correlation between NLR/PLR, HE, and poor outcomes after ICH, thereby investigating the clinical value of these markers in the early prediction of HE.

## 2. Materials and Methods

### 2.1. Study Population

**Figure 1:**
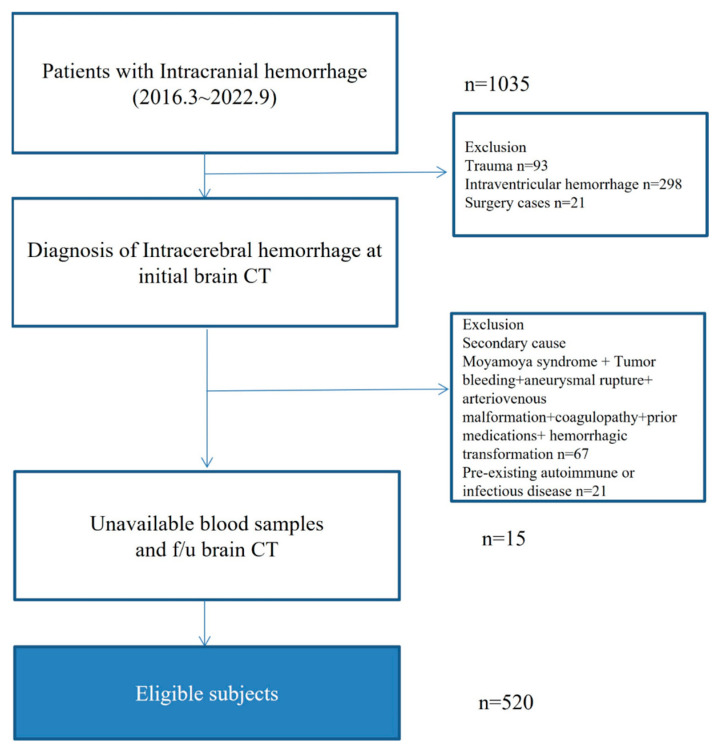
Flow chart of this study.

We consecutively registered all patients with acute spontaneous ICH who were hospitalized at Chuncheon Sacred Heart Hospital between March 2016 and September 2022. All patients with ICH underwent brain computed tomography (CT) immediately after hospitalization at the emergency department of our institution (Figure 1). Exclusion criteria for this study included age under 18, surgical cases on hospitalization, ICH secondary to a brain tumor, trauma, Moyamoya syndrome, aneurysmal rupture, arteriovenous malformation, coagulopathy, previous anticoagulation therapy, drug abuse, hemorrhagic transformation after ischemic stroke, immunomodulatory management before ICH, and all individuals with pre-existing autoimmune or infectious diseases. We strictly controlled the blood pressure of enrolled patients in the intensive care unit, a stroke predictor for HE, according to the American Heart Association’s recommendations [14].

### 2.2. Data Collection

We retrieved the following data from all enrolled patients from our prospective registry database: (1) demographics (age and sex), (2) medical history (alcohol use, prior stroke, hypertension, diabetes mellitus, hyperlipidemia, atrial fibrillation, current smoking status, and pre-stroke medication), (3) stroke characteristics (acute management of ICH, initial Glasgow coma scale (GCS) score, initial National Institute of Health Stroke Scale (NIHSS) score and lesion location of ICH), (4) laboratory parameters (hemoglobin, white blood cell count, neutrophil count, platelet count, lymphocyte count, serum creatinine, initial random glucose, low-density lipoprotein, prothrombin time, glycated hemoglobin, and blood pressure).

Whole blood samples were collected upon arrival at the emergency department. This study’s mean time interval from arrival to blood sampling was 22.3 min (±5.4). The samples were centrifuged (2000 rpm for 20 min at 4 °C) immediately after collection in a calcium ethylenediaminetetraacetic acid tube. Subsequently, cell counts were analyzed using the same auto-analyzer (XE-2100, Sysmex, Kobe, Japan). NLR was calculated by classifying the absolute neutrophil count by the absolute lymphocyte count. PLR was calculated by dividing the absolute platelet count by the absolute lymphocyte count.

The primary outcome measure was HE, defined as an absolute growth greater than 6 mL or a relative growth of more than 33% in the follow-up brain CT to the initial brain CT [15]. In our institutional protocol, we performed routine follow-up brain CT at 24 h and 72 h after ICH onset or at the time of detecting neurological deterioration. The ICH volume was estimated using the widely used ABC/2 method [16]. The two expert neurologists (C Kim and S-H Lee) assessed all ICH volumes in a double-blind manner (interclass correlation coefficient = 0.89). The secondary outcome measures were poor functional outcome at 3 months (modified Rankin Scale (mRS) score ≥ 3) and 1-month mortality. Poor functional outcomes were defined as an mRS > 3 [17].

### 2.3. Statistical Analysis

Variables were compared using Pearson’s Chi-squared test for categorical variables and Student’s *t*-test or the Mann–Whitney U test for continuous variables concerning the presence of HE. The independent effects of initial NRL and PLR values on the outcome measures were evaluated using binary logistic regression analysis. Variables with *p*-values < 0.1 and clinically plausible outcomes, when associated with NRL and PLR values, were adjusted in the multivariate analysis. Additionally, we calculated the crude and adjusted odds ratios (ORs) and 95% confidence intervals (CIs).

We performed a receiver operating characteristic (ROC) curve using the ‘pROC’ package of R to assess the predictive ability of NLR and PLR levels on HE. Delong’s test calculated the 95% CI for the area under the curve (AUC) and *p*-value. NLR and PLR cut-off values for HE were calculated using the Youden index.

The impact of NLR and PLR tertiles on HE and other outcomes was evaluated using multivariate sensitivity analysis.

Statistical analyses were performed using IBM SPSS version 21.0 software (IBM Corporation, Armonk, NY, USA) and R version 4.0.3 (R core Team 2020; R Foundation for Statistical Computing, Vienna, Austria).

## 3. Results

A total of 520 patients with ICH were enrolled in this study (mean age of 64.2 ± 15.7; 60% male). Among these patients, 91 (17.5%) had HE within 72 h of ICH onset. The baseline ICH volume was 25.5 ± 33.9 mL. The HE group was likely older, less likely to be male, and had more severe initial neurological symptoms, hypertension, diabetes mellitus, higher glycated hemoglobin level, initial random glucose level, initial systolic blood pressure (SBP), and SBP at follow-up CT than the no-HE group (mean time from initial CT to follow-up CT: 11.4 ± 5.7 h). Median NLR and PLR values in the HE group were higher than those in the no-HE group (Table 1). Brain CT angiography (CTA) was not routinely performed after hospitalization; hence, some patients (*n* = 72) had missing spot sign data. After excluding patients with missing CTA data, spot signs were not significantly different in the HE and no-HE groups (10.5% vs. 14.7%, *p* = 0.31, respectively).

**Figure 2:**
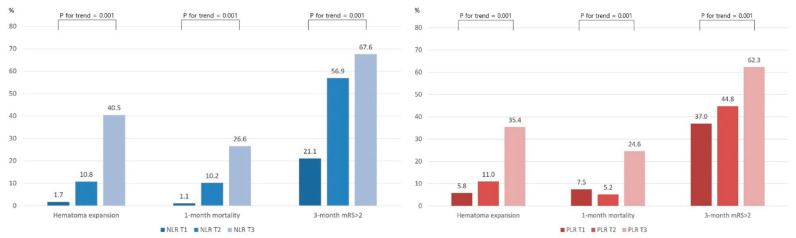
Comparing clinical outcomes according to NLR and PLR tertiles. Abbreviation: NLR, neutrophil–lymphocyte ratio; PLR, platelet–lymphocyte ratio; mRS, modified Rankin Scale; T1, lowest tertile; T2, middle tertile; T3, highest tertile.

The occurrence of HE showed a significantly increasing trend with a higher tertile of NLR. Moreover, 1-month mortality and proportion of 3-month mRS of 3–6 also showed an increasing trend with a higher tertile of NLR. Similarly, HE occurrence, 1-month mortality, and 3-month mRS of 3–6 showed an increasing trend with a higher tertile of PLR (Figure 2).

In the multivariate analysis, NLR was associated with an increased occurrence of HE (OR [95% CI], 1.19 [1.12–1.27], *p* < 0.001, Table 2 and Supplementary Table S1). In addition, NLR increased the chance of 1-month mortality and poor functional outcomes at 3 months (Table 3 and Supplementary Table S2). Similarly, PLR was associated with an increased occurrence of HE (OR [95% CI], 1.01 [1.00–1.02], *p* = 0.04; Table 2 and Supplementary Table S1). However, PLR was not associated with 1-month mortality or poor functional outcomes at 3 months (Table 3). As a sensitivity analysis, the higher NLR tertiles were associated with HE, 1-month mortality, and 3-month poor functional outcome. Moreover, PLR tertiles were associated with HE and 1-month mortality but not with the 3-month poor functional outcome (Table 2 and Table 3, Supplementary Tables S3 and S4).

**Figure 3:**
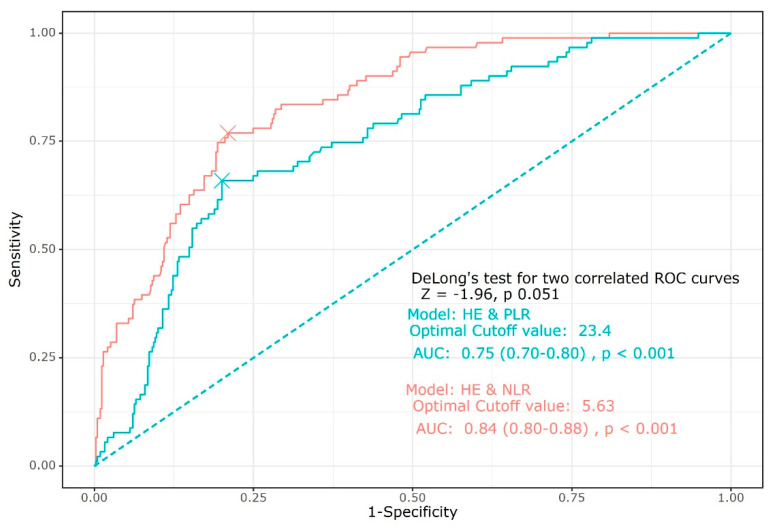
Receiver operating characteristics curves of NLR and PLR for predicting hematoma expansion. Abbreviation: NLR, neutrophil–lymphocyte ratio; PLR, platelet–lymphocyte ratio.

The ROC curve showed that the predictive abilities of both NLR and PLR were close to good (AUC of NLR: 0.84, 95% CI [0.80–0.88], *p* < 0.001; AUC of PLR: 0.75 95% CI [0.70–0.80], *p* < 0.001). There were no significant differences in the HE prediction between NLR and PLR. The cut-off value of NLR for predicting HE was 5.63, and that of PLR was 23.4 (Figure 3).

## 4. Discussion

The main findings in this study were as follows: (1) the values of NLR and PLR were higher in the HE group than in the no HE group; (2) both higher NLR and PLR increased the risk of HE after acute ICH in multivariate analysis; (3) the ROC curve showed that both NLR and PLR were reliable for predicting HE, and the cut-off values of NLR and PLR were 5.63 and 23.4 respectively.

Our main findings showed that higher NLR increased the risk of HE, and sensitivity results were robust to our main results. It has been well documented that inflammatory reactions could be associated with HE, thereby leading to pathologic propagation after acute ICH [18,19]. However, the clinical relationship between acute inflammatory reaction and HE remains controversial because of inconsistent results reported in previous studies [8,10]. When ICH occurs, neutrophils are known as the first inflammatory cells to invade the brain parenchyma [20]. Therefore, NLR could be a complex index that reflects systemic inflammatory status [21] and was recently identified as a novel marker for predicting several outcomes after ICH [22]. Nonetheless, evidence of the predictive value of NLR for HE in patients with acute ICH was still lacking [23,24,25]. Although activated neutrophil in the acute stage of ICH may biologically promote a procoagulant state and limit HE [26,27], elevating NLR could promote neurotoxicity and altered permeability of the blood–brain barrier and vessel integrity, thereby ameliorating HE and brain edema in acute ICH [28]. Additionally, secondary brain injury and cellular DNA damage via the expression of several pro-inflammatory cytokines could explain the short-term and long-term poor outcomes after ICH [29,30,31,32]. We carefully proposed that our results could support the aforementioned pathological evidence and showed the usefulness of NLR in the acute setting of ICH.

In addition to the inflammatory state, a hypercoagulable state should also be implicated in patients with ICH. An acute inflammatory reaction could promote hypercoagulation and subsequent thrombosis, leading to a vicious inflammation and thrombosis cycle [33,34]. Platelets induce the release of inflammatory cytokines and exacerbate the inflammatory process [35]. Moreover, the PLR reflects the status of coagulation and inflammation and could be a potential risk factor for the hyperinflammatory process [36,37,38]. We assumed that PLR could be a reliable predictor for HE in patients with ICH, as PLR provides accurate inflammatory and thrombotic status [38]. Nonetheless, a few studies evaluated the association between PLR and outcomes after ICH [11,39], and our study is the first to evaluate the impact of PLR on HE after ICH. Interestingly, higher PLR increases the risk of HE and other worse short- and long-term outcomes after ICH. One previous study, including 247 participants, reported that increased PLR values could be associated with poor outcomes in patients with subarachnoid hemorrhage and ICH [40]. In addition, previous studies reported that higher PLR increased the risk of mortality, complications, and initial severe neurological symptoms in patients with ICH [11,13,39]. Our main results could provide robust evidence and were generally consistent with previous results. However, some studies have shown contradicting perspectives of a negative association between higher PLR and worse outcomes. An observational study (*n* = 170) reported that lower PLR could be an independent factor for higher survival in patients with traumatic brain injury [41]. That phenomenon explains that spontaneous aggregation and excessive platelet consumption after brain injury induce platelet depletion, thereby breaking the balance of coagulation and anticoagulation, enhancing bleeding risk. In addition, a higher tertile of PLR was not associated with worse outcomes at 3 months, although raw PLR could increase the risk in this study. A previous meta-analysis showed that the timing of the acquisition of PLR (early or delayed) might affect outcomes and that early PLR could not be associated with 3-month outcomes [42]. Further studies are warranted to evaluate the association between platelet-mediated inflammatory reactions and thrombosis according to the phases of ICH.

Given the devastating impact of HE on disability and mortality after ICH, early prediction and identification of HE are crucial. The NLR and PLR could be feasible markers for predicting HE in real-world practice. We could easily and rapidly calculate the NLR and PLR using one blood sample. Although patients had several physiological and pathological conditions, these ratios were stable indices. Hence, the usefulness of NLR and PLR was evaluated in several diseases, such as cancer, heart failure, and infectious disease [43,44,45]. Moreover, the combined utilization of NLR and PLR values reflects the pro-inflammatory and procoagulant status after ICH; hence, it was rational to predict HE in the acute phase of ICH using a combination of both ratios. In addition, the reliable predictability of NLR and PLR in our results could help establish treatment strategies in acute ICH settings. A previous study on ischemic stroke treated with reperfusion therapy reported that both NLR/PLR could predict hemorrhagic transformation [46,47]. However, our results using NLR/PLR are novel for predicting HE. Although there were strong predictors for HE after ICH [48], we carefully proposed that the combined utilization of NLR/PLR could aid early HE prediction by blood sampling.

This study has several limitations. First, although we consecutively collected data from the registry database, the data could have a retrospective nature. Second, since early blood sampling was performed once without serial measurements, the association between serial changes in NLR/PLR and outcomes after ICH could not be determined. However, because this study aimed to evaluate the impact and reliability of NLR/PLR on HE in the early stage, serial NLR/PLR measurements could be inevitable. Third, although several variables were adjusted in the multivariate analysis, the generalization of this study’s results could be hindered by unmeasured confounding factors. Fourth, other inflammatory markers activated in cases of acute ICH were unavailable in this study.

## 5. Conclusions

This study showed that a higher NLR and PLR significantly increased HE risk after the acute phase of ICH. Given that NLR and PLR are simple and easily available markers, the joint application of both indices may be helpful for clinicians in predicting HE and performing appropriate management for high-risk patients. Further large-scale and well-designed studies are necessary to confirm this study’s results.

## Figures and Tables

**Table 1:** Comparison baseline characteristics between HE and no-HE groups.

	Hematoma Expansion (−) *n* = 429	Hematoma Expansion (+) *n* = 91	*p*-Value
Age, year (SD)	62.8 (15.9)	70.8 (12.5)	0.02
Male, *n* (%)	266 (62.0)	46 (50.5)	0.04
Interval from onset to sample, hour (IQR)	4 (2–6)	4 (2–6)	0.32
Initial NIHSS, score (IQR)	5 (4–10)	16 (10–20)	<0.001
Initial GCS, score (IQR)	13 (8–15)	5 (4–9)	<0.001
Hypertension, *n* (%)	224 (52.2)	59 (64.8)	0.04
Diabetes mellitus, *n* (%)	88 (20.5)	33 (36.3)	0.002
Hyperlipidemia, *n* (%)	35 (8.2)	9 (9.9)	0.68
CAD, *n* (%)	29 (6.8)	9 (9.9)	0.37
Current smoking, *n* (%)	47 (11.0)	10 (11.1)	0.90
Prior stroke, *n* (%)	72 (16.8)	19 (20.9)	0.36
Prior antiplatelet use, *n* (%)	119 (27.7)	29 (31.9)	0.44
Prior anticoagulation, *n* (%)	14 (3.3)	7 (7.7)	0.07
ICH lesion, *n* (%)			0.13
deep	182 (42.4)	31 (34.1)	
lobar	172 (40.1)	39 (42.9)	
infratentorial	51 (11.9)	18 (19.8)	
Combined IVH	24 (5.6)	3 (3.3)	
WBC count, ×10^9^/L (SD)	11.25 (23.21)	16.51 (48.21)	0.08
Platelet count, ×10^9^/L (SD)	215.1 (94.3)	233.8 (100.7)	0.22
Hemoglobin, g/L (SD)	13.7 (2.0)	12.4 (2.2)	0.26
Prothrombin time, INR (IQR)	1.05 (1.01–1.13)	1.07 (1.01–1.19)	0.16
LDL, mg/dL (SD)	87.8 (34.9)	73.8 (34.9)	0.49
Creatinine, mg/dL (SD)	1.1 (2.3)	1.4 (1.6)	0.12
CRP, mg/dL (SD)	12.2 (27.9)	15.1 (33.0)	0.14
HbA1c, % (SD)	6.0 (1.0)	6.3 (1.2)	0.01
Initial random glucose, mg/dL (SD)	147.9 (55.2)	202.1 (83.9)	<0.001
Initial SBP, mmHg (SD)	157.9 (32.7)	162.8 (40.0)	0.01
SBP at f/u CT, mmHg (SD)	111.9 (15.8)	119.0 (20.3)	<0.001
DBP, mmHg (SD)	78.3 (18.4)	80.8 (19.7)	0.06
NLR, (SD)	3.08 (1.77–5.16)	8.24 (5.80–14.41)	<0.001
PLR, (SD)	11.76 (6.08–21.17)	29.05 (14.75–41.51)	<0.001
Initial ICH volume, mL (IQR)	9 (3–25)	24 (9–81)	<0.001
Hemostatic therapy, *n* (%)	90 (21.0)	25 (27.9)	0.21

Abbreviation: HE, hematoma expansion; SD, standard deviation; IQR, interquartile range; NIHSS, National Institute of Health Stroke Scale; GCS; Glasgow Coma Scale; CAD, Coronary artery disease; ICH, intracerebral hemorrhage; IVH, intraventricular hemorrhage; WBC, white blood cell; INR, international normalized ratio; LDL, low-density lipoprotein; CRP, C-reactive protein; HbA1c, glycated hemoglobin; SBP, systolic blood pressure; CT, computed tomography; f/u; follow up; DBP, diastolic blood pressure; NLR, Neutrophil–lymphocyte ratio; PLR, platelet–lymphocyte ratio.

**Table 2:** Multivariate analysis showing the impact of NLR and PLR on hematoma expansion.

	OR *	95% CI	*p*-Value		OR *	95% CI	*p*-Value
Raw NLR	1.19	1.12–1.27	<0.001	Raw PLR	1.01	1.00–1.02	0.04
NLR T1	reference			PLR T1	reference		
NLR T2	3.12	0.82–11.83	0.10	PLR T2	2.33	0.93–5.80	0.07
NLR T3	19.96	5.58–71.34	<0.001	PLR T3	6.30	2.77–14.34	<0.001

Abbreviation: NLR, Neutrophil–lymphocyte ratio; PLR, platelet–lymphocyte ratio; OR, odds ratio; CI, confidence interval; T1, lowest tertile; T2, middle tertile; T3, highest tertile. * Adjusting age, male, hypertension, diabetes mellitus, prior anticoagulation, initial GCS, initial NIHSS, WBC, HbA1c, initial random glucose, initial SBP, SBP at f/u CT, and initial ICH volume.

**Table 3:** Multivariate analysis showing the impact of NLR and PLR on clinical outcomes.

	1-Month Mortality						3-Month mRS 3–6				
	OR *	95% CI		OR *	95% CI		OR *	95% CI		OR *	95% CI
Raw NLR	1.16	1.08–1.24	Raw PLR	1.01	0.99–1.02	Raw NLR	1.10	1.05–1.26	Raw PLR	0.995	0.98–1.01
NLR T1	reference		PLR T1	reference		NLR T1	reference		PLR T1	reference	
NLR T2	3.09	0.49–19.37	PLR T2	0.76	0.21–2.71	NLR T2	3.46	1.56–7.67	PLR T2	1.62	0.79–3.33
NLR T3	13.23	2.24–78.33	PLR T3	3.04	1.07–8.59	NLR T3	3.24	1.45–7.25	PLR T3	0.89	0.40–1.98

Abbreviation: NLR, Neutrophil–lymphocyte ratio; PLR, platelet–lymphocyte ratio; mRS, modified Rankin Scale; OR, odds ratio; CI, confidence interval; T1, lowest tertile; T2, middle tertile; T3, highest tertile. * Adjusting age, male, hypertension, diabetes mellitus, prior anticoagulation, initial GCS, initial NIHSS, WBC, HbA1c, initial random glucose, initial SBP, SBP at f/u CT, and initial ICH volume.

## Supplementary Materials

The following supporting information can be downloaded at: https://www.mdpi.com/article/10.3390/jcm12083004/s1.
