# Supplementary material for: The Clinical Value of Neutrophil-to-Lymphocyte Ratio and Platelet-to-Lymphocyte Ratio for Predicting Hematoma Expansion and Poor Outcomes in Patients with Acute Intracerebral Hemorrhage
Source: J Clin Med. 2023 Apr 20;12(8):3004. doi: 10.3390/jcm12083004 (PMC10145379; doi:10.3390/jcm12083004)
Supplement: Supplementary file 1 [file jcm-12-03004-s001.zip › jcm-2308540-supplementary.pdf]

Supplementary Table S1. Multivariate analysis showing that impact of NLR and PLR on hematoma expansion.

	OR	95% CI	p-value		OR	95% CI	p-value
NLR	1.19	1.12-1.27	<0.001	PLR	1.01	1.00-1.02	0.04
Age	1.02	0.99-1.04	0.09	Age	1.03	1.00-1.05	0.02
Male	0.86	0.43-1.71	0.67	Male	0.96	0.55-1.98	0.90
Hypertension	1.08	0.56-2.06	0.82	Hypertension	1.23	0.67-2.27	0.5
Diabetes mellitus	1.45	0.66-3.19	0.36	Diabetes mellitus	1.56	0.76-3.21	0.23
Prior anticoagulation	2.95	0.95-9.16	0.06	Prior anticoagulation	2.63	0.86-7.99	0.09
Initial GCS	0.74	0.59-0.94	0.02	Initial GCS	0.78	0.62-0.98	0.03
Initial NIHSS	0.99	0.85-1.14	0.87	Initial NIHSS	1.02	0.89-1.18	0.77
WBC	1.00	0.99-1.01	0.90	WBC	0.996	0.99-1.00	0.32
HbA1c	1.20	0.88-1.64	0.25	HbA1c	1.03	0.76-1.39	0.85
Initial random glucose	1.01	1.00-1.01	0.04	Initial random glucose	1.01	1.00-1.01	0.003
Initial SBP	1.01	0.99-1.02	0.39	Initial SBP	1.00	0.99-1.01	0.73
SBP at f/u CT	1.01	0.98-1.03	0.62	SBP at f/u CT	1.00	0.99-1.01	0.85
Initial ICH volume	0.99	0.99-1.01	0.75	Initial ICH volume	1.00	1.99-1.01	0.85

Abbreviation: NLR, Neutrophil-lymphocyte ratio; PLR, platelet-lymphocyte ratio; OR, odd ratio; CI, confidence interval; GCS, Glasgow coma scale; NIHSS, National Institute of Health Stroke Scale; WBC, white blood cell; HbA1c, glycated hemoglobin; SBP, systolic blood pressure; f/u, follow up; CT, computed tomography; ICH, intracranial hemorrhage

Supplementary Table S2. Multivariate analysis showing that impact of raw NLR and raw PLR on clinical outcomes.

	1-month mortality					3-month mRS 3-6					
	OR	95% CI		OR	95% CI		OR	95% CI		OR	95% CI
NLR	1.16	1.08-1.24	PLR	1.01	0.99-1.02	NLR	1.10	1.05-1.26	PLR	0.995	0.98-1.01
Age	1.07	1.03-1.11	Age	1.07	1.03-1.10	Age	1.40	1.02-1.07	Age	1.05	1.02-1.07
Male	1.83	.68-4.95	Male	1.74	0.69-4.39	Male	1.10	0.56-2.18	Male	1.2	0.62-2.34
HTN	1.50	0.61-3.73	HTN	1.38	0.59-3.24	HTN	0.44	0.23-0.86	HTN	0.48	0.25-0.91
DM	1.53	0.50-4.66	DM	1.70	0.61-4.75	DM	3.40	1.53-7.54	DM	3.59	1.63-7.92
Prior anticoagulation	7.79	1.46-41.55	Prior anticoagulation	6.17	1.23-3.09	Prior anticoagulation	0.83	0.21-2.24	Prior anticoagulation	0.88	0.24-3.23
Initial GCS	0.63	0.43-0.93	Initial GCS	0.73	0.51-1.04	Initial GCS	0.64	0.48-0.85	Initial GCS	0.67	0.51-0.89
Initial NIHSS	1.16	0.95-1.42	Initial NIHSS	1.21	0.99-1.47	Initial NIHSS	0.64	0.48-0.85	Initial NIHSS	1.25	0.99-1.57
WBC	1.04	0.95-1.13	WBC	1.04	0.98-1.11	WBC	1.06	1.01-1.10	WBC	1.06	1.01-1.12
HbA1c	0.88	0.55-1.41	HbA1c	0.79	0.51-1.23	HbA1c	1.09	0.74-1.61	HbA1c	1.02	0.70-1.50
Initial random glucose	1.01	1.00-1.02	Initial random	1.01	1.00-1.02	Initial random glucose	1.01	1.00-1.02	Initial random	1.01	1.00-1.02

			glucose						glucose		
Initial SBP	1.01	0.99-1.02	Initial SBP	1.00	0.99-1.02	Initial SBP	0.99	0.98-1.01	Initial SBP	0.99	0.98-1.01
SBP at f/u CT	1.02	0.99-1.05	SBP at f/u CT	1.03	1.00-1.06	SBP at f/u CT	1.02	0.99-1.05	SBP at f/u CT	1.02	0.996-1.05
Initial ICH volume	1.00	0.99-1.01	Initial ICH volume	1.00	0.99-1.01	Initial ICH volume	0.99	0.98-1.01	Initial ICH volume	0.997	0.98-1.01

Abbreviation: NLR, Neutrophil-lymphocyte ratio; PLR, platelet-lymphocyte ratio; mRS, modified Rankin Scale; OR, odd ratio; CI, confidence interval; HTN, hypertension; DM, diabetes mellitus; GCS, Glasgow coma scale; NIHSS, National Institute of Health Stroke Scale; WBC, white blood cell; HbA1c, glycated hemoglobin; SBP, systolic blood pressure; f/u, follow up; CT, computed tomography; ICH, intracranial hemorrhage

Supplementary Table S3. Multivariate analysis showing the impact of tertiles of NLR on outcomes after ICH.

	HE			1-month mortality			3-month mRS 3-6		
	OR	95% CI	p-value	OR	95% CI	p-value	OR	95% CI	p-value
NLR T1		reference			reference			reference	
NLR T2	3.12	0.82-11.83	0.10	3.09	0.49-19.37	0.23	3.46	1.56-7.67	0.002
NLR T3	19.96	5.58-71.34	<0.001	13.23	2.24-78.33	0.004	3.24	1.45-7.25	0.004
Age	1.03	1.00-1.05	0.03	1.07	1.03-1.11	<0.001	1.05	1.02-1.07	<0.001
Male	0.94	0.47-1.88	0.85	1.92	0.70-8.27	0.20	1.35	0.67-2.70	0.40
Hypertension	1.06	0.55-2.06	0.86	1.30	0.54-3.14	0.56	0.49	0.25-0.95	0.03
Diabetes mellitus	1.56	0.70-3.50	0.28	1.80	0.58-5.58	0.31	2.85	1.28-6.37	0.01
Prior anticoagulation	1.61	0.48-5.35	0.44	3.45	0.69-17.36	0.13	1.01	0.25-4.13	0.99
Initial GCS	0.75	0.58-0.86	0.02	0.68	0.47-0.99	0.04	0.66	0.49-0..88	0.01
Initial NIHSS	0.997	0.85-1.16	0.97	1.18	0.96-1.45	0.11	1.22	0.96-1.54	0.10
WBC	1.00	0.99-1.01	0.99	1.04	0.96-1.12	0.35	1.05	1.01-1.10	0.03
HbA1c	1.21	0.88-1.68	0.25	0.85	0.53-1.39	0.52	1.15	0.78-1.70	0.49
Initial random glucose	1.01	1.00-1.01	0.06	1.01	1.00-1.02	0.01	1.01	1.00-1.02	0.04
Initial SBP	1.00	0.99-1.02	0.66	1.00	0.99-1.02	0.83	0.99	0.98-1.01	0.20
SBP at f/u CT	1.01	0.99-1.03	0.43	1.03	0.998-1.06	0.07	1.02	0.998-1.05	0.07
Initial ICH volume	0.997	0.99-1.01	0.46	0.999	0.99-1.01	0.86	0.99	0.98-1.01	0.36

Abbreviation: NLR, Neutrophil-lymphocyte ratio; ICH, intracranial hemorrhage; HE, hematoma expantion; mRS, modified Rankin Scale; OR, odd ratio; CI, confidence interval; T1, lowest tertile; T2, middle tertile; T3, highest tertile; GCS, Glasgow coma scale; NIHSS, National Institute of Health Stroke Scale; WBC, white blood cell; HbA1c, glycated hemoglobin; SBP, systolic blood pressure; f/u, follow up; CT, computed tomography

Supplementary Table S4. Multivariate analysis showing the impact of tertiles of PLR on outcomes after ICH.

	HE			1-month mortality			3-month mRS 3-6		
	OR	95% CI	p-value	OR	95% CI	p-value	OR	95% CI	p-value
PLR T1		reference			reference			referene	
PLR T2	2.33	0.93-5.80	0.07	0.76	0.21-2.71	0.67	1.62	0.79-3.33	0.19
PLR T3	6.30	2.77-14.34	<0.001	3.04	1.07-8.59	0.04	0.89	0.40-1.98	0.77
Age	1.02	0.998-1.05	0.07	1.06	1.03-1.10	0.001	1.05	1.02-1.08	<0.001
Male	0.92	0.47-1.80	0.82	1.53	0.58-4.05	0.39	1.24	0.63-2.43	0.54
Hypertension	1.25	0.67-2.34	0.48	1.31	0.55-3.11	0.54	0.46	0.23-0.88	0.02
Diabetes mellitus	1.52	0.71-3.24	0.28	1.50	0.53-4.26	0.45	3.81	1.72-8.46	0.001
Prior anticoagulation	2.24	0.73-6.95	0.16	5.05	0.96-26.47	0.06	0.94	0.25-3.55	0.93
Initial GCS	0.74	0.58-0.94	0.01	0.68	0.47-0.99	0.046	0.67	0.50-0.88	0.01
Initial NIHSS	0.99	0.86-1.15	0.92	1.17	0.96-1.44	0.13	1.24	0.99-1.56	0.06
WBC	0.998	0.99-1.01	0.62	1.04	0.96-1.12	0.32	1.06	1.01-1.12	0.01
HbA1c	1.12	0.82-1.52	0.49	0.89	0.56-1.41	0.61	1.05	0.71-1.55	0.82
Initial random glucose	1.01	1.00-1.01	0.02	1.01	1.00-1.02	0.003	1.01	1.00-1.02	0.02
Initial SBP	0.998	0.99-1.01	0.79	0.999	1.98-1.02	0.90	0.99	0.98-1.01	0.31
SBP at f/u CT	1.01	0.99-1.03	0.26	1.03	1.00-1.06	0.053	1.02	0.998-1.05	0.08
Initial ICH volume	0.999	0.99-1.01	0.85	1.00	0.99-1.01	0.76	0.997	0.98-1.01	0.70

Abbreviation: PLR, Platelet-lymphocyte ratio; ICH, intracranial hemorrhage; HE, hematoma expantion; mRS, modified Rankin Scale; OR, odd ratio; CI, confidence interval; T1, lowest tertile; T2, middle tertile; T3, highest tertile; GCS, Glasgow coma scale; NIHSS, National Institute of Health Stroke Scale; WBC, white blood cell; HbA1c, glycated hemoglobin; SBP, systolic blood pressure; f/u, follow up; CT, computed tomography
